# Sacubitril/Valsartan: Potential Impact of ARNi “Beyond the Wall” of ACE2 on Treatment and Prognosis of Heart Failure Patients With Coronavirus Disease-19

**DOI:** 10.3389/fcvm.2020.616564

**Published:** 2020-11-27

**Authors:** Speranza Rubattu, Giovanna Gallo, Massimo Volpe

**Affiliations:** ^1^Cardiology Unit, Department of Clinical and Molecular Medicine, School of Medicine and Psychology, Sant'Andrea Hospital, Sapienza University of Rome, Rome, Italy; ^2^Istituto di Ricovero e Cura a Carattere Scientifico Neuromed, Pozzilli, Italy

**Keywords:** COVID-19, natriuretic peptide, ARNi, cardiovascular diseases, HFrEF—heart failure with reduced ejection fraction

## Introduction

From the beginning of the SARS-CoV-2 pandemia, the type 2 angiotensin-converting enzyme (ACE2), probably the most “unloved and neglected” member of the renin-angiotensin-aldosterone (RAAS) family, has attracted increasing attention since it has been shown as the cell receptor through which the virus enters into the cells (1).

The physiological action of ACE2, a membrane protein expressed in the heart, lungs, kidneys, liver, and intestine, consists in degrading angiotensin II (Ang II) to angiotensin (1-7), a heptapeptide with a potent vasodilator function through the Mas receptor able to counterbalance the Ang II effects on vasoconstriction, sodium retention, and fibrosis (1). Previous studies have shown that Ang II type 1 receptor (AT1R) blockers (ARBs), ACE inhibitors (ACEI), and mineralocorticoid receptor antagonists (MRA) may up-regulate the expression of ACE2 both in acute and chronic settings of cardiovascular diseases (CVDs), such as hypertension, heart failure (HF) and myocardial infarction (1). These data have generated concern during the early phases of the pandemia, since it has been speculated that the increase in ACE2 level may have contributed to disease virulence and to adverse outcomes particularly in subjects affected by chronic coexisting conditions, namely hypertension, coronary artery disease, HF, and diabetes, who commonly received treatment with RAAS inhibitors and who were characterized by a worse clinical course (2).

On the other hand, it has been observed that the binding between coronavirus and ACE2 leads to ACE2 downregulation, resulting in an unopposed production of Ang II by ACE, contributing to lung damage as a consequence of AT1R mediated inflammation, fibrosis, thrombosis, vasoconstriction, and increased vascular permeability. According to these findings, RAAS inhibitors and, in particular, ARBs may even protect against COVID-19 acute lung injury (1). As a matter of fact, epidemiological studies conducted in large populations of COVID-19 patients demonstrated that ARBs or ACE inhibitors had no association with a severe or fatal course of the disease (3–5).

## Evidence Supporting the Potential Beneficial Role of ARNi in HF Patients With COVID-19

Natriuretic peptides (NPs), which include atrial natriuretic peptide (ANP), brain natriuretic peptide (BNP), and C-type natriuretic peptide (CNP), along with their N-terminal counterparts, may play an important protective role in COVID-19 disease. NPs are released as a consequence of increased volume overload and myocytes stress and, through their vasorelaxant, diuretic, and effects, are able to counterbalance RAAS and sympathetic nervous system actions, ultimately regulating blood pressure, electrolytes, and water homeostasis (6). At the vascular level, NPs reduce cellular growth and proliferation, preserving endothelial function and integrity as well as vascular tone, and they oppose blood clotting, inflammation, angiogenesis, and atherosclerosis progression (6). Apart from their well-described systemic hemodynamic and autocrine/paracrine functions within the cardiovascular system, NPs also play an important protective role in the lungs. In fact, ANP reduces lung endothelial permeability caused by inflammation and oxidative stress, avoiding the development of acute respiratory distress syndrome and improving arterial oxygenation during mechanical ventilation (7). According to this evidence, it has been proposed that COVID-19 patients with deficiencies in the NP system, mainly obese subjects and black people, may have an increased risk of developing severe lung complications.

Of interest, a bidirectional interaction between NPs, particularly ANP, and ACE2 has been demonstrated in experimental models. ANP, through cyclic guanosine monophosphate (cGMP) production, inhibited the Ang II-mediated activation of the extracellular signal regulated kinase (ERK1/ERK2) pathway and upregulated the mitogen-activated protein kinase phosphatase (MKP1), finally preventing the decrease in ACE2 mRNA synthesis (8). On the other hand, Ang-(1-7), the product of ACE2 activity, stimulated ANP secretion through the Mas receptor/phosphatidylinositol 3-kinase/protein kinase B (Mas/PI3K/Akt) pathway, thus reducing cardiac hypertrophy and fibrosis and potentially avoiding COVID-19 pulmonary damage (8).

Furthermore, consistently with the well-known prognostic role of NPs, it has been demonstrated that NT-proBNP level represents an independent risk factor of in-hospital death in patients with severe COVID-19, its levels being significantly higher among those patients who experienced severe clinical conditions, and increasing further during hospitalization in subjects who died, without significant changes among survivors (9).

Apart from the known pathogenetic, diagnostic, and prognostic implications in the cardiovascular system (10), NPs have relevant therapeutic properties. In this context, a field of great interest may be represented by the potential impact on the clinical course of the COVID-19 disease and on its outcome of a treatment with sacubitril/valsartan (S/V), a member of the new pharmacological class of AT1R/neprilysin inhibitors (ARNi). S/V is now recognized as a cornerstone of the therapeutic management of HF with reduced ejection fraction (HFrEF) due to the impressive benefits on cardiovascular death and HF hospitalization (11).

The beneficial effects of S/V in HFrEF were confirmed in recent real-life clinical studies showing a significant reduction of cardiac death and HF rehospitalization, an improvement of echocardiographic parameters, such as left ventricular EF, systolic volume, and systolic pulmonary arterial pressure, of renal function and of quality of life (12–14). Moreover, S/V treatment can be safely started during hospitalization in daily clinical practice with no evidence of increased risk of hypotension, worsening of renal function and hyperkalaemia (15).

With regard to the trend of different NPs levels after the initiation of S/V, NT-proBNP level decreases as a consequence of the improvement of cardiac function and haemodynamic status, representing a useful biomarker of treatment response; BNP level slightly increases due to its relatively low affinity to neprilysin, whereas ANP level consistently and substantially increases both in human studies and in experimental models, mediating most of the benefits of neprilysin inhibition (16, 17).

According to these evidences, an approach based on early administration of S/V has been proposed in the therapeutic management of all COVID-19 hospitalized patients to avoid an adverse clinical course (18).

## Perspectives

**Figure 1:**
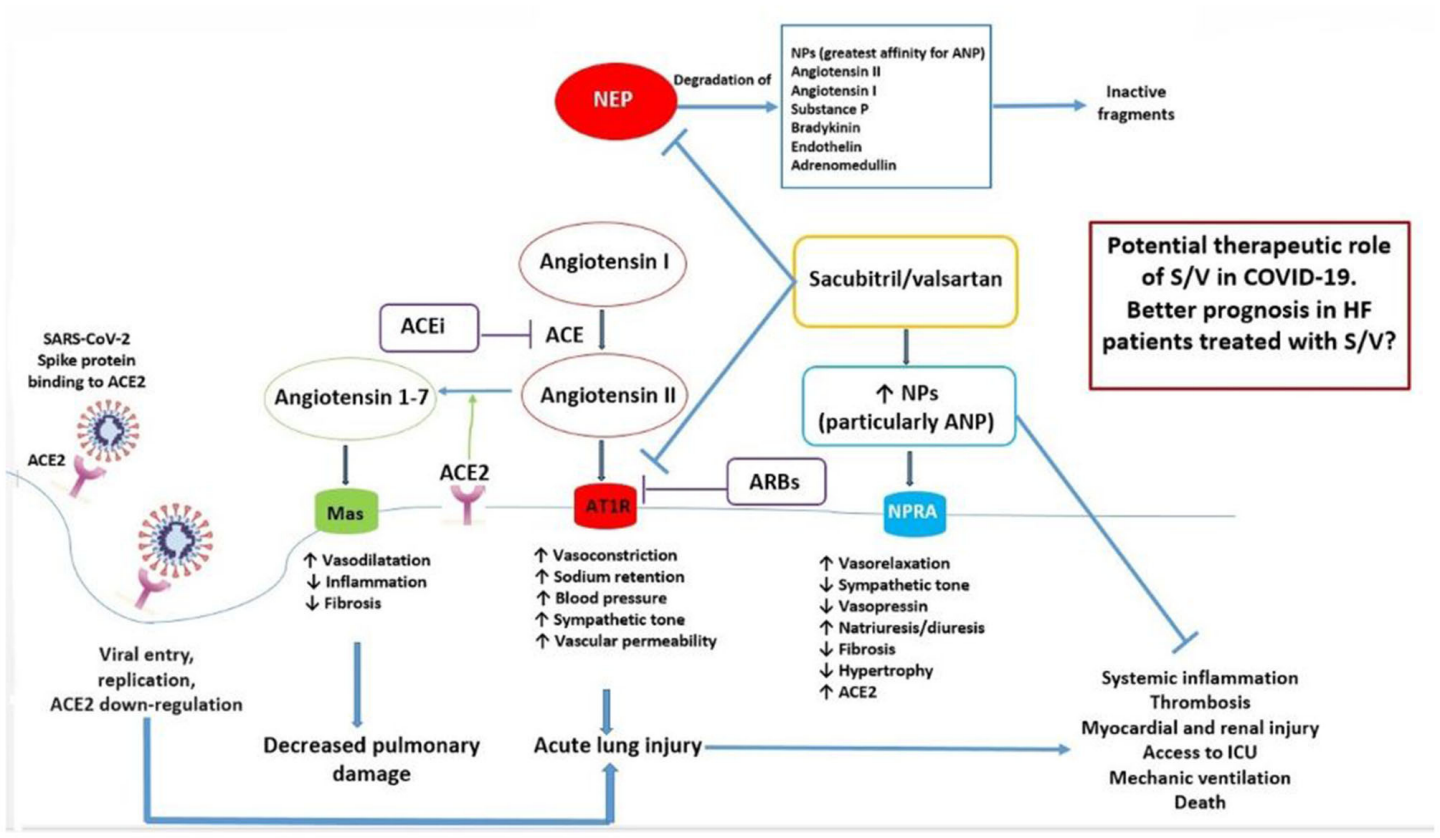
Mechanisms underlying the potential beneficial effects of sacubitril/valsartan in HF patients with COVID-19. Ang 1-7, produced by ACE2 from Ang II, and NPs, particularly ANP, may protect from acute lung injury, systemic inflammation, and adverse outcomes during SARS-CoV-2 infection. On the other hand, local activation of the RAAS system may mediate injury responses to viral insults. S/V inhibits both the AT1R and neprilysin, which degrades NPs. As a consequence, S/V may exert an important protective function from adverse clinical course, mediated by an increase of ANP and by AT1R blockade, in HF patients with COVID-19. ACE, angiotensin converting enzyme; ACE2, type 2 angiotensin converting enzyme; ACE-i, ACE inhibitor; ANP, atrial natriuretic peptide; ARB, angiotensin type I receptor blocker; AT1R, angiotensin type I receptor; HF, heart failure; ICU, intensive care unit; NEP, neprilysin; NPs, natriuretic peptides; NPR-A, natriuretic peptide receptor A; S/V, sacubitril/valsartan.

Based on the ability of S/V to increase ANP level while antagonizing the Ang II/AT1R effects, we propose a major protective role of this class of drugs in HFrEF patients, the only current indication for the use of ARNi, when affected by COVID-19 disease (Figure 1). In order to test the expected beneficial role of S/V in COVID-19, a retrospective analysis of existing registries of hospitalized COVID-19 patients could help to find out whether, among subjects affected by HFrEF, those who were already treated with S/V presented a lower disease incidence, better prognosis, and clinical course (particularly in terms of intensive care unit access, mechanical ventilation, and death), compared to patients who received other medications, including ACEI/ARBs. Furthermore, a call to action is requested to test the potential benefits of S/V in HFrEF patients affected by COVID-19 through new prospective randomized clinical trials.

## Author Contributions

SR, GG, and MV contributed to the conception and design, acquisition of data, or analysis and interpretation of data, drafted the article, and approved the final version to be published. All authors contributed to the article and approved the submitted version.

## Conflict of Interest

The authors declare that the research was conducted in the absence of any commercial or financial relationships that could be construed as a potential conflict of interest.
